# Kenaf Core as an Alternative Soilless Growing Medium: A Review

**DOI:** 10.3390/plants15040666

**Published:** 2026-02-23

**Authors:** Conner C. Austin, S. Brooks Parrish, David G. Clark, Ann C. Wilkie

**Affiliations:** 1 Horticultural Sciences Department, University of Florida-IFAS, Gainesville, FL 32611, USA; brooks.parrish@ufl.edu (S.B.P.); geranium@ufl.edu (D.G.C.); 2 Department of Soil, Water, and Ecosystem Sciences, University of Florida-IFAS, Gainesville, FL 32611, USA

**Keywords:** soilless culture, container substrates, horticultural sustainability, coco coir, peat

## Abstract

Kenaf (*Hibiscus cannabinus*) core, an abundant renewable byproduct rich in cellulose and hemicellulose, has emerged as a candidate to replace or supplement peat and coco coir in soilless culture. This review synthesizes the physical, chemical, and biological performance of ground kenaf core and benchmarks it against conventional substrates. Kenaf core exhibits low bulk density (0.06 to 0.15 g cm^−3^), high total porosity (approximately 90%), and substantial plant available water (approximately 42%), supporting root aeration and water supply. Its pH (6.0–7.2) is near optimal for most crops, whereas electrical conductivity (EC) (3.2–4.7 dS m^−1^) can exceed recommended ranges for salt-sensitive species, which necessitates pre-leaching or blending. Growth studies show comparable shoot and root performance in blends containing 20 to 70% kenaf, with composted kenaf often outperforming raw core. Pure kenaf generally requires more frequent irrigation and may shrink at high proportions. We outline processing variables such as core purity, particle size, composting, and leaching that govern stability and plant response, identify critical data gaps (including standardized EC and pH methods, and long-term shrinkage), and frame a sustainability agenda. Practically, studies to date indicate that pre-leached kenaf core, incorporated at up to about 70% by volume into peat or coir-based blends with structurally stable components such as perlite, can maintain growth and quality for several ornamental and bedding crops under greenhouse and nursery conditions. At the same time, reports of poor performance in some conifers and early suppression in direct-sown vegetables underscore that the suitability of kenaf-based substrates remains crop specific and dependent on material processing and management.

## 1. Introduction

The science of growing media has evolved significantly since the mid-20th century, with soilless substrates now dominating containerized plant production due to their ability to promote vigorous root growth, enable efficient nutrient uptake, and mitigate risks from soilborne pathogens [1,2]. However, the industry’s reliance on two primary components, peat and coco coir, is facing a confluence of critical challenges [3]. Peat is increasingly viewed as ecologically unsustainable due to its slow regeneration rate and the damage that extraction inflicts on vital carbon sinks [4,5]. This has prompted intensifying political initiatives to reduce or eliminate its use, creating regulatory urgency for high-performing alternatives such as wood fiber and compost [6]. At the same time, while coco coir is a renewable byproduct, life cycle assessments confirm that the majority of its environmental footprint stems from the impacts of long-distance sea transport from tropical production zones [7]. Compounding these environmental and political pressures are significant supply chain disruptions, including cost volatility and interconnected barriers of availability, affordability, and performance consistency that affect everyone from producers to consumers [8,9,10,11]. Taken together, these factors underscore a pressing need to identify and validate a new class of renewable, locally producible materials that are not only scientifically sound but also economically viable and logistically practical.

Recent work has synthesized the broader landscape of peat alternatives, including wood fiber and other organic byproducts, using formal systematic review methods. For example, Sdao et al. [11] provide a comprehensive assessment of multiple candidate materials in relation to peat reduction targets. In contrast, the present review adopts a deliberately focused scope and concentrates on ground kenaf core as a single, underutilized substrate component. By collating and benchmarking the dispersed literature on kenaf’s physical, chemical, biological, and crop performance characteristics, we aim to provide a depth of material-specific analysis that complements these broader evaluations of peat substitutes.

In this context, kenaf (*Hibiscus cannabinus*) is a promising renewable ingredient for soilless media. A fast-growing annual suitable for cultivation in many warm-season production regions, its central stalk comprises an outer bast (35%) and an inner core (65%) [12]. The bast fiber is well suited to applications such as paper pulp and biocomposites due to its high cellulose content and fiber length [13,14,15,16,17,18,19]. The woody inner core is often treated as a byproduct, yet its high hemicellulose content and absorbency make it useful for filtration aids, oil absorbents, and as a component of growing media [4,17,19,20,21,22,23]. Early studies show that as a partial replacement in peat-based blends, ground kenaf core delivers comparable performance for many crops, although this varies with species, particle size, and blend ratio.

Existing studies indicate that ground kenaf core can, under certain conditions, support plant growth comparable to conventional peat- and coir-based substrates, particularly when used in blends. However, the available evidence is fragmented across crops and regions, and important aspects of substrate performance such as standardized physical and chemical characterization, long-term structural stability, and quantitative sustainability metrics remain incompletely described or inconsistently reported. In this review, we therefore (i) synthesize published data on the physical, chemical, and biological properties of kenaf core in soilless systems, (ii) benchmark these properties and crop responses against peat and coco coir, and (iii) identify specific knowledge gaps and research priorities needed to assess when and how kenaf can function as a reliable substrate component. By framing what is known, what is uncertain, and what must be measured next, we aim to provide a clear basis for future experimental work and for evidence-based evaluation of kenaf core within modern soilless culture.

## 2. Review Methodology

This review was conducted as a narrative, topic-focused synthesis rather than a systematic review; nevertheless, we followed a structured approach to identify and select relevant literature. Primary peer-reviewed studies evaluating kenaf (*Hibiscus cannabinus*) core as a component of soilless substrates were identified using Google Scholar, with searches conducted between November 2024 and December 2025. Search queries combined terms such as “kenaf core growing media”, “kenaf potting medium”, “kenaf container substrate”, and “kenaf soilless culture”, along with related phrases such as “peat alternative”, “coconut coir”, and “container substrate physical properties”. Reference lists of key articles were also screened (backward citation searching) to capture additional relevant studies.

We focused on studies that (i) reported the use of ground or composted kenaf core or whole-stem kenaf as a component of container media, (ii) provided quantitative data on plant growth, substrate physical or chemical properties, or both. We excluded studies that used kenaf exclusively for non-horticultural applications (such as for biocomposites and paper pulp) unless they provided compositional data directly relevant to substrate performance. Contextual information on peat and coco coir, as well as global trade patterns in growing media constituents, was obtained from peer-reviewed articles, extension publications, and international databases. In particular, trade statistics for peat and coco coir were extracted from the UN Comtrade database (https://comtrade.un.org, accessed on 15 December 2025) using the relevant HS codes.

Because the body of literature specifically addressing kenaf core as a growing medium is relatively limited and spans multiple decades, we did not restrict our search to a fixed publication window; instead, we included all accessible studies up to December 2025 that met the above criteria. Where possible, we prioritized original experimental work over secondary summaries, and we synthesized findings across studies to highlight consistent patterns as well as knowledge gaps in the physical, chemical, biological, and sustainability dimensions of kenaf-based substrates.

## 3. Desirable Characteristics of Growing Media

To support plant health, growing media must perform four core functions: provide physical support, aerate the root zone, supply water, and deliver nutrients [24,25]. A substrate’s ability to perform these functions is determined by its physical, chemical, and biological properties. These characteristics are interdependent and dictate a medium’s suitability for a given crop and production system [24,25]. However, since reported values for these properties depend heavily on test protocols, standardized methods and clear reporting are essential for meaningful comparisons. This review therefore uses these three property classes as the framework for comparing kenaf core against peat and coco coir.

### 3.1. Physical Properties

The physical properties of a growing medium are central to healthy root develop- ment and sustained plant growth. A high-quality substrate maintains a balanced relationship between air and water, which enables root respiration, nutrient uptake, and overall plant vigor [26,27]. These properties originate from the size, shape, and packing arrangement of the substrate’s particles and are commonly described by metrics including particle size distribution, bulk density, and total porosity. These foundational properties in turn determine functional outcomes such as water-holding capacity and aeration [24,25,26].

Particle size distribution governs a substrate’s pore architecture and therefore its porosity and bulk density. For many container systems, a particle size range of 0.25 to 2.5 mm provides a desirable balance of air-filled porosity and water retention [25,28]. Bulk density, the mass of medium per unit volume, is inversely related to total porosity. Lower bulk density, with typical values for soilless mixes falling near 0.2 to 0.5 g cm^−3^, indicates a looser structure that promotes aeration and root expansion. Higher bulk density suggests compaction that can restrict air movement and plant growth [25,29,30,31]. Total porosity, the fraction of volume occupied by pores, usually ranges from 50 to 90% by volume in effective mixes [24,25,30,31,32,33].

Water-holding capacity is the fraction of pore space that remains filled with water after gravitational drainage, while aeration is the air-filled fraction in the same state [24]. The two are inversely related; as total porosity increases, aeration generally increases while water-holding capacity decreases. For many crops in small to medium containers, a water-holding capacity of about 50 to 60% and an air-filled porosity of 20 to 30% are common targets. However, optimal values always vary with container height, irrigation regime, and specific crop requirements [25,30,34].

In summary, a substrate’s key physical properties are tightly linked and must be optimized in concert to provide a root environment that supports robust growth. Because reported values can vary with test protocols, container dimensions, and handling, the clear documentation of measurement methods is critical for comparing results across studies.

### 3.2. Chemical Properties

A substrate’s chemical properties govern nutrient availability at the root interface. The key indicators are pH, electrical conductivity (EC), and cation exchange capacity (CEC), which growers can adjust through practices like liming, fertigation, and pre-leaching to optimize plant growth [26,35,36]. Because reported values for these metrics depend on the test method and extraction ratio, clear documentation is essential for meaningful comparisons [24,25,26].

The pH, which indicates the acidity or alkalinity of the substrate solution, strongly influences nutrient solubility and availability [24]. At high pH, the availability of phosphorus and several micronutrients decline. Conversely, at low pH, aluminum and manganese can become phytotoxic and the availability of key macronutrients may be reduced [25,37,38]. Most container crops perform best within a pH range of 5.2 to 6.5, which ensures broad nutrient availability [25,30,31].

Electrical conductivity measures the concentration of soluble salts in the root-zone solution and serves as a proxy for fertility status [25,39]. While moderate EC indicates adequate nutrients, excessive EC can cause osmotic stress and reduce water uptake, and very low EC suggests nutrient deficiency. Although target ranges are method and crop dependent, many soilless systems aim for an EC between 1.5 and 3.5 dS m^−1^, with lower targets for salt-sensitive species [25,34].

Cation exchange capacity is the substrate’s ability to adsorb and exchange positively charged ions such as K^+^, Ca^2+^, and Mg^2+^, providing a buffer and reservoir for nutrients [25,40]. For organic soilless media, a CEC between 50 and 200 cmolc kg^−1^ is often desirable, as it promotes nutrient retention without excessive fixation. Substrates with very low CEC are prone to rapid leaching and unstable nutrition, while very high CEC can slow fertilizer adjustments and contribute to imbalances if not managed correctly [25,35,41].

In summary, pH, EC, and CEC jointly regulate nutrient supply and overall plant performance. Optimizing substrates and enabling valid comparisons across studies requires managing these properties within crop-specific targets and consistently reporting the measurement methods used.

### 3.3. Biological Properties

A substrate’s biological properties strongly influence its performance and ability to support healthy plant development [26,42]. The key factors are the presence of harmful organisms, the material’s biological stability, and the potential for nutrient immobilization. Each requires active management to maintain a favorable root environment [26]. Pathogens, pests, and weed seeds can severely damage plant health by causing root rots, fungal infections, and direct root damage that impairs water and nutrient uptake [26,43,44,45]. To minimize these risks, high-quality media must be produced using clean sourcing, screening, and sanitation. Pasteurization or steam treatment is often preferred over complete sterilization because it reduces pathogen loads while preserving beneficial microflora that aid in nutrient cycling and disease suppression. Similarly, preventing weed seed contamination reduces competition for resources and helps ensure uniform crop growth [26,44].

Biological stability is critical for organic substrates, which are subject to microbial decomposition. As microorganisms break down carbon-rich components, the medium can shrink, reducing air-filled porosity and impairing root respiration [46,47]. This decomposition can also alter water relations, sometimes increasing water retention to the point of waterlogging if irrigation is not adjusted [26,48]. Stability can be managed by selecting appropriate particle sizes, blending with structurally stable components, and using pretreatments like composting to reduce the most degradable fractions. High microbial activity can also temporarily immobilize nutrients, particularly nitrogen and phosphorus, as microorganisms consume these ions while metabolizing carbon [26,42]. This process can lead to transient nutrient deficiencies and slow plant growth. To counteract this, growers must monitor substrate nutrient levels and provide supplemental fertilization as needed to maintain adequate nutrition throughout the crop cycle. Managing a substrate’s biological profile is integral to its performance. By preventing contamination, maintaining structural stability, and offsetting nutrient immobilization through best practices, growers can sustain a healthy root environment that minimizes risks and supports consistent, high-quality plant growth.

## 4. Commonly Utilized Growing Media

Peat and coco coir are the dominant substrates in containerized plant production, a status supported by massive international trade flows (Table 1). To illustrate the scale, 2023 peat imports to the United States exceeded 1.1 million metric tons at a value of nearly USD 500 million, which was part of a global trade valued at over USD 1.9 billion [49]. While the market for coco coir is smaller, it remains significant, with global imports valued at over USD 462 million in the same year [49]. These figures underscore the horticultural sector’s deep reliance on imported substrates and its vulnerability to supply chain disruptions and price volatility. The primary exporters of peat are Latvia, Estonia, Germany and Canada, while those of coco coir are India and Sri Lanka, resulting in substantial transport distances to supply large markets such as the United States.

### 4.1. Peat

Peat remains the dominant substrate in containerized production because it combines high CEC, excellent water retention, and favorable aeration. Together, these properties support robust root growth and provide a strong nutrient buffer [2,25,26,50,51,52]. Derived from partially decomposed mosses like sphagnum that accumulate in bogs, peat typically exhibits a low bulk density (0.04 to 0.2 g cm^−3^), high total porosity (83.8 to 96.4%), and a high CEC (90 to 140 cmolc kg^−1^) [36,52,53,54]. It also has a low inherent EC of around 0.2 to 0.49 dS m^−1^ [25,52].

However, peat presents significant practical and environmental challenges. Its natural acidity, with a typical pH near 3.9, requires liming for most crops [52]. It also becomes hydrophobic once dry, making it difficult to rewet uniformly and increasing the risk of crop stress [3]. Beyond the greenhouse, peat extraction is increasingly scrutinized for its environmental impact. Peatlands cover only 3% of the global land surface but are vital carbon sinks and hotspots of biodiversity [55,56,57]. Harvesting peat degrades these functions and habitats, raising questions about its long-term sustainability. These environmental concerns, combined with the costs of extraction, processing, and long-distance transport, are driving the search for alternatives. While peat’s physical and buffering properties make it a valuable substrate, there is a clear and growing need to substitute it with sustainable, locally sourced materials that can maintain high performance.

### 4.2. Coco Coir

Coco coir (also called coco coir pith or coco peat), a byproduct derived from the fibrous coconut mesocarp, is another widely used substrate component [58]. It offers several advantages, including a near-optimal pH (5.6 to 6.9) that rarely requires adjustment and excellent rewetting properties. Its physical characteristics are also favorable, with a low bulk density (0.04 to 0.08 g cm^−3^) and high total porosity (85.5 to 89.5%) [52,59]. Coco coir has a higher EC compared to peat, with values ranging from 0.3 to 2.9 dS m^−1^ [59]. However, its CEC (39 to 60 cmolc kg^−1^) is considerably lower than that of peat [59].

Coco coir’s primary challenges are high salt content and inconsistent quality. Material sourced from coastal regions often contains elevated levels of sodium, chloride, and potassium that can impair plant growth unless it is aged and thoroughly washed or buffered [58,60]. The process of creating horticultural-grade coir can take up to two years, leading to significant variability in EC and nutrient profiles between suppliers and even between batches. This inconsistency adds processing costs and management complexity for growers. Although often positioned as a sustainable alternative to peat, these issues of quality control, lengthy processing, and the costs associated with freight from tropical production zones can limit coir’s practical utility. Its successful use hinges on sourcing reliable, pre-treated products and using regular substrate tests to guide fertility management.

## 5. Kenaf as Growing Media

The dried, ground core of the kenaf plant is a promising material for use in growing media. Research has demonstrated its value both as an amendment and as a complete substrate. As an additive in conventional mixes, kenaf core can improve aeration and root development, with performance comparable to peat-based blends at moderate inclusion rates [23,61]. As a complete replacement for traditional media, its performance is more variable and highly dependent on careful management of irrigation and fertility, as well as on pretreatments like composting or leaching [4,23,62].

However, wider adoption of kenaf core is constrained by limited data and a lack of standardized testing protocols. To move forward, research must focus on establishing a more complete and comparable profile of the material. Key priorities include quantifying its long-term structural stability and shrinkage under commercial irrigation, characterizing its nutrient release dynamics, and determining its CEC using standardized methods. Furthermore, given reports of high EC in some batches, best-practice guidelines for pre-leaching and blending are needed. Defining optimal processing variables, such as core purity, particle size, and composting protocols, will ultimately enable growers to realize kenaf’s full potential as a sustainable and cost-effective substrate.

### 5.1. Growth Studies

Growth studies across a range of vegetables and ornamentals support that ground kenaf core is a valuable component in soilless mixes, though performance hinges on inclusion rate, particle size, and crop species (Table 2). For example, when used as an additive for tomato (*Lycopersicon esculentum*), a blend with 20 to 35% finely ground kenaf core in peat yielded the best overall shoot growth [61]. A broader study evaluating a 70% kenaf and 30% peat mix across 17 species showed more nuanced results. While root mass was generally enhanced in the kenaf-amended media, shoot dry weight was higher in the peat control for seven species and comparable for the remaining ten [63]. Reinforcing this theme of variability, Webber et al. [22] found that when kenaf core was used to substitute pine bark and vermiculite, its effectiveness depended entirely on the grind fineness, blend proportion, and the specific crop being grown. This underscores the need to tailor kenaf blends to a particular application.

Ground kenaf core has also been tested as a complete replacement for conventional substrates, but results suggest it performs best in blends. For instance, Wang [4] found that while several ornamentals could grow in 100% kenaf core, they required substantially more irrigation. Blends containing 70 to 80% kenaf, however, produced shoot growth comparable to or better than commercial mixes. Williams and Reichert [23] reported a similar pattern: slower growth in pure kenaf but comparable performance in a 70% kenaf and 30% peat mix. Performance is also species-specific, as Tsakaldimi [62] found that kenaf-based media performed poorly for *Pinus halepensis* seedlings, suggesting some species, like conifers, may be less suited to kenaf substrates.

Beyond performance variability, some studies report early growth suppression, particularly when whole-stem kenaf (core plus bark) is used at sowing. In a study on lettuce and pepper, even a low 10% ratio of whole-stem kenaf to sand reduced seedling height, leaf number, and biomass [64]. Although this inhibition was partially alleviated by soaking the kenaf in ammonium nitrate, normal growth resumed only after seedlings were transplanted into kenaf-free media. These findings underscore the importance of material processing and suggest that for sensitive species, kenaf is best introduced at transplant rather than at germination.

Pretreatments like composting can also significantly improve performance. Laiche and Newman [65] reported that *Ilex crenata* cv. Cherokee grown in composted kenaf core compared favorably to plants grown in a standard pine bark medium. Overall, the evidence indicates that ground kenaf core is most effective as a component in blends, typically at up to 70% by volume. Its performance is optimized by selecting an appropriate particle size, pre-leaching to reduce high EC, and using composted material to enhance stability and mitigate potential growth inhibition.

Taken together, these studies span a range of climatic and production contexts, from humid subtropical regions of the southern United States (Texas, Mississippi) to temperate greenhouse environments (Delaware, Oklahoma) and Mediterranean conditions in Greece. These differences in climate and production systems likely influence irrigation demand, substrate drying cycles, and the rate of kenaf core decomposition, all of which can affect crop response. For example, in warmer, more humid regions, higher evapotranspiration and more frequent irrigation may accentuate shrinkage and structural changes in kenaf-based media, whereas in cooler or more controlled environments, the same mixes may remain physically stable for longer crop cycles. Nevertheless, across these diverse locations, a consistent pattern emerges: kenaf core performs best as a component of blended substrates rather than as a sole medium, suggesting that the general conclusions of this review are broadly applicable across production regions, provided that irrigation and fertility are locally optimized.

### 5.2. Physical Properties of Kenaf as Growing Media

Ground kenaf core exhibits favorable physical characteristics that are comparable to both peat and coco coir (Table 3). Its bulk density, at 0.06 to 0.15 g cm^−3^, is similar to peat and indicates a light, loose structure that resists compaction [22,52,62]. Its total porosity has been reported at 90.7%, a value which aligns with ranges for peat and coir and ensures ample pore space for root aeration [62]. Furthermore, its plant available water content is also high at approximately 41.9%, placing it within the upper range of conventional substrates and suggesting it can supply effective moisture to the root zone [62].

Together, these properties demonstrate that ground kenaf core can provide a suitable physical environment for root growth in soilless systems. It is important to note, however, that reported values for porosity and available water can vary significantly depending on the test protocol, container dimensions, and packing method used. For this reason, clear documentation of measurement approaches is essential for valid comparisons across different studies and materials.

In summary, the physical properties of ground kenaf core, as reported to date, fall broadly within the same general range as those of peat and coco coir for parameters such as bulk density, total porosity, and plant available water. Given the variability in measurement protocols among studies, these comparisons should be interpreted as indicative rather than as demonstrating strict equivalence. Within this context, and with appropriate particle sizing and pretreatment, kenaf core appears capable of providing a suitable root environment and functioning as a substrate component in blends that can reduce reliance on imported materials.

### 5.3. Chemical Properties of Kenaf as Growing Media

The chemical characteristics of ground kenaf core are promising, though they also highlight areas where further research is needed. Its most significant advantage is a near-optimal pH, which typically ranges from 6.03 to 7.17 (Table 4) [4]. This is comparable to coco coir and falls within the preferred range for most container crops, reducing or eliminating the need for liming that is required for acidic peat-based media [25,30,31].

In contrast, kenaf’s EC presents a management challenge. Reported EC values of 3.18 to 4.7 dS m^−1^ are considerably higher than those of peat or coir and can exceed the recommended targets for salt-sensitive species [4]. This makes pre-leaching or blending with lower-EC components a necessary practice for many applications. As with other properties, reported EC values are highly dependent on the measurement method and extraction ratio, so consistent reporting is critical for cross-study comparisons.

Ground kenaf core contains measurable amounts of several essential mineral nutrients. For instance, one study reported total calcium and magnesium concentrations (12 and 2.34 mg g^−1^, respectively) that are considerably higher than typical values for peat and coir (Table 5) [62]. The core’s nitrogen content (1.44%) was also found to be comparable to peat and higher than coir. These values suggest that kenaf core may contribute to the baseline supply of several key nutrients, although differences in analytical methods and the limited number of reports mean that these comparisons with peat and coir should be viewed as approximate. However, these figures represent total elemental content, not the plant-available fraction. The release kinetics of these nutrients under typical fertigation regimes remain uncharacterized. Therefore, while kenaf has the potential to supplement crop nutrition and perhaps reduce initial fertilizer inputs, existing evidence does not justify reducing fertigation inputs without careful monitoring. In practice, it is prudent to base fertility management on regular substrate testing rather than to assume a substantial nutrient contribution from the kenaf itself.

To more reliably characterize the chemical performance of ground kenaf core, a targeted research agenda on its chemical behavior is needed that uses standardized analytical methods. This agenda should prioritize three key areas. First, its nutrient dynamics must be characterized using standardized measurement protocols for CEC and EC, along with a thorough analysis of nutrient release kinetics over time, so that future comparisons with peat and coir are based on directly comparable data. Second, the long-term stability of its pH and EC must be assessed under commercial growing conditions. To support this, all studies must consistently report measurement methods, such as saturated paste or pour-through, to ensure results are comparable. Finally, the effects of pretreatments like composting and leaching should be systematically evaluated to quantify their impact on salt levels and potential phytotoxic compounds. By generating this body of method-rich, comparable data, kenaf core can be optimized for widespread adoption as a sustainable and efficient growing medium.

### 5.4. Biological Properties of Kenaf as Growing Media

The biological properties of kenaf core are primarily defined by its chemical composition: approximately 32.1% cellulose, 41% hemicellulose, and 25.2% lignin (Table 6). Its lignin content is significantly lower than that of coco coir and is at the lower end of the range reported for peat. Because lignin provides structural rigidity and resists microbial breakdown, kenaf’s lower lignin content makes it more susceptible to decomposition than more stable substrates [25].

This higher rate of decomposition has two major consequences for growers. The first is volumetric shrinkage, which reduces total porosity and air-filled pore space, potentially impairing root respiration. This effect is most pronounced at high inclusion rates, with one study reporting significant shrinkage when kenaf exceeded 70% of the mix by volume [4]. The second consequence is the potential for nutrient release as microorganisms break down organic matter. While this can contribute to plant nutrition, it may also lead to imbalances if not monitored.

Managing these biological properties is key to the successful use of kenaf. Shrinkage can be mitigated by blending kenaf with more structurally stable components like perlite, managing particle size, or using composted core, which has already undergone initial decomposition. To manage nutrient dynamics, monitoring substrate pH and EC and adjusting fertigation accordingly appear important, particularly in kenaf-rich mixes where decomposition and salt levels may change over time. Further research is needed to quantify shrinkage rates under commercial irrigation and to define optimal particle size and pretreatment protocols to maximize long-term stability.

**Figure 1:**
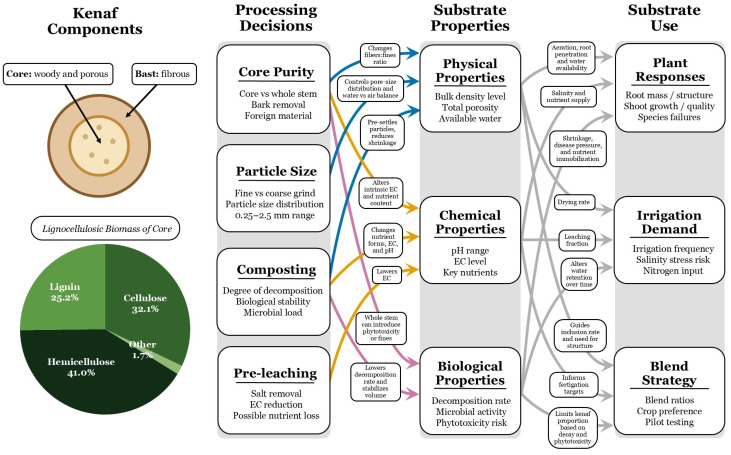
***Conceptual process–property–performance framework for ground kenaf core in soilless substrates.*** The figure highlights that kenaf core performance emerges from the interaction of structure, processing, and management rather than from any single factor. The leftmost panel illustrates key structural and compositional features of kenaf core (high hemicellulose and cellulose, lower lignin, high porosity and low bulk density) [18] that underlie its behavior as a growing medium component. Processing decisions (core purity, particle size, composting, pre-leaching) modify these features through specific mechanisms (for example, altering pore-size distribution and water–air balance, stabilizing or accelerating decomposition and shrinkage, or removing soluble salts and reducing EC). These changes in physical, chemical, and biological properties in turn influence plant responses (root development, shoot growth, species-specific failures), irrigation and fertigation demand, and blend strategy. Colored arrows indicate processing-driven mechanisms affecting substrate properties: blue arrows highlight changes that primarily alter physical properties, orange arrows highlight mechanisms that primarily affect chemical properties, and pink arrows highlight mechanisms affecting biological properties. Gray arrows represent downstream impacts of these properties for plant responses, irrigation demand, and blend strategy.

Taken together, the studies reviewed here suggest a process–property–performance framework for kenaf-based substrates (Figure 1). Processing decisions such as core purity (removal of bark and extraneous stem tissue), particle size distribution, and the use of composting and pre-leaching directly influence key physical and chemical properties, including bulk density, porosity and available water, shrinkage behavior, pH, and EC. These property shifts, in turn, help explain the observed variation in plant responses, such as differences in root mass, shoot growth, irrigation demand, and sensitivity to salinity across species and production systems. Finally, management choices at the grower level, including blend ratio with peat or coir, the inclusion of structurally stable components like perlite, and irrigation and fertigation regimes, can be used to moderate these effects and align kenaf-based media with crop-specific requirements. This framework provides a basis for interpreting the diverse results summarized in Table 2, Table 3, Table 4, Table 5 and Table 6 as parts of a coherent substrate system rather than as isolated findings.

Notwithstanding these promising indications, the current evidence base on kenaf core as a substrate component has important limitations. Most growth studies have been conducted on a relatively narrow set of ornamental and bedding crops under greenhouse or nursery conditions in a limited number of geographic regions, and documented failures in species such as *Pinus halepensis* and in direct-sown lettuce and pepper show that responses can be strongly crop- and management-specific. Structurally, the relatively low lignin content and high hemicellulose fraction raise concerns about decomposition and volumetric shrinkage at high inclusion rates, particularly under frequent irrigation. Chemically, high native EC in some batches constrains use for salt-sensitive species unless effective pre-leaching is implemented, and long-term trajectories of pH and EC under commercial fertigation regimes remain poorly quantified. Economically and environmentally, potential advantages related to local cultivation and dual-use of bast and core are still hypothetical in the absence of kenaf-specific life-cycle and cost analyses. Addressing these limitations through standardized characterization, long-term performance trials across diverse environments, and quantitative sustainability assessments will be essential to define where kenaf-based substrates are truly fit for purpose and where alternative materials may be more appropriate.

## 6. Discussion

The search for sustainable growing media has become urgent as producers confront the environmental, economic, and logistical drawbacks of relying on peat and coco coir [9,25]. In this context, ground kenaf core emerges as a credible, locally sourced candidate substrate component. The studies synthesized here indicate that, when properly managed and used in appropriate blends, kenaf core can supplement conventional substrates without compromising crop performance for several tested ornamental and bedding species [4,22,23,61,62,63,65,66]. However, these findings also indicate that the performance of kenaf core is constrained by crop- and system-specific responses, potential shrinkage at high inclusion rates, and uncertainties about its long-term chemical behavior and sustainability.

The evidence presents a mixed but interpretable profile of benefits and constraints. Physically, reported values for kenaf core’s bulk density and total porosity fall broadly within the same order of magnitude as those reported for peat [52] and coco coir [59], and its near-neutral pH [4,62] reduces or eliminates the need for liming [25,30,31]. At the same time, some lots exhibit relatively high native EC that often requires pre-leaching [4], and the lower lignin content [18] may increase susceptibility to microbial decomposition and volumetric shrinkage, particularly at high inclusion rates [4,46,47,48]. Growth studies in ornamental and bedding crops suggest that blends containing roughly 20 to 70% kenaf core by volume can produce shoot and root growth similar to commercial mixes under greenhouse and nursery conditions [4,22,23,61,63,65,66], when particle size, irrigation, and fertility are adjusted appropriately. In contrast, species such as *Pinus halepensis* and direct-sown lettuce and pepper have performed poorly in kenaf-containing media under the reported conditions [62,64], which underscores that kenaf is not a universally suitable substrate component and that its use must be evaluated on a crop- and system-specific basis. More generally, because the reported physical and chemical properties of kenaf, peat, and coco coir derive from heterogeneous measurement protocols, the numerical comparisons presented here should be interpreted as indicative of broad ranges rather than as standardized benchmarks that demonstrate strict equivalence among substrates.

From a sustainability perspective, kenaf core has several potential advantages that warrant further quantitative evaluation. As a renewable annual crop, kenaf can be cultivated in many warm-season production regions, which may reduce the transport distances and associated emissions compared with imported substrates such as peat and coco coir [4,5,7,8,50,51,52]. In addition, the dual-use model in which the bast fiber serves higher-value industrial applications (paper pulp, biocomposites), leaving the core as a byproduct for horticultural use, suggests a possible economic and resource-efficiency benefit. However, robust kenaf-specific life cycle or carbon footprint data for growing media applications are not yet available, so these putative advantages remain to be demonstrated formally. Realizing and documenting any such benefits at scale will require not only optimized processing infrastructure and quality assurance but also dedicated life-cycle assessments that compare kenaf-based substrates with conventional mixes under consistent functional units.

For practice, a cautious implication of the current evidence is that pre-leached and, where feasible, composted kenaf core can function as an amendment in blends with peat or coir for certain crops, particularly ornamentals and bedding plants, under greenhouse and nursery conditions similar to those in the published studies [4,22,23,61,62,63,65,66]. Performance depends on tailoring the particle size and blend ratio to the crop and production system, and on managing irrigation to account for the water-holding characteristics and EC of kenaf-based mixes. Regular monitoring of substrate pH and EC remains essential, and it would be advisable to evaluate new formulations at small scale before broader adoption.

Despite these potential benefits, there is a notable lack of quantitative life-cycle assessment data specific to kenaf core used as a growing medium. Existing assessments of growing media focus mainly on peat, coco coir, or other organic constituents and do not consider kenaf core within a comparable functional unit, such as per cubic meter of substrate or per crop cycle. As a result, robust indicators such as global warming potential, cumulative energy demand, and water use cannot yet be directly compared between kenaf-based substrates and conventional peat- or coir-based mixes. This absence of kenaf-specific life-cycle data represents an important research gap and currently limits the ability to rigorously quantify the environmental benefits that are suggested by its agronomic performance and supply chain characteristics.

To transition kenaf from a niche alternative to a mainstream component, a targeted research roadmap is required. This roadmap must prioritize three areas: (i) Standardization, including universal methods for measuring and reporting CEC, EC, and pH to ensure cross-study comparability; (ii) Performance optimization, through studies that quantify long-term shrinkage and determine optimal particle sizes and pretreatment protocols for representative crops; and (iii) Economic validation, via life-cycle assessments that clarify the cost and carbon savings of kenaf relative to conventional substrates.

In conclusion, ground kenaf core shares many of the essential attributes of a high-quality growing medium and appears to be a promising candidate substrate component. Current evidence suggests that, when pre-treated and used in appropriate blends, kenaf core can help reduce reliance on non-renewable peat and globally shipped coco coir for certain crops and production systems. At the same time, further work is needed to standardize methods for physical and chemical characterization, quantify long-term structural stability and shrinkage, and generate kenaf-specific life-cycle and economic assessments. With these research needs addressed, kenaf-based substrates could more reliably support high-quality crop production while contributing to a more sustainable and resilient future for soilless culture.

## Figures and Tables

**Table 1:** United States and global imports of peat and coco coir in 2023, quantity (t) and trade values (USD). Source: UN Comtrade, 2023 [49].

Commodity	Net Weight (t)		Approximate Value (Million $)	
	USA	Global	USA	Global
Peat	1,172,104	8,036,966	494	1900
Coco Coir	75,738	1,105,622	52	462

**Table 2:** Summary of published growth studies evaluating ground kenaf core as an additive or replacement in soilless substrates, with species tested, main outcomes, and references.

Species	Main Outcomes	Location	Reference
*Lycopersicon esculentum* (Tomato)	Finely ground kenaf core at 20 to 35% in peat gave best shoot growth vs. other ratios; supports use as additive at moderate inclusion.	Newark, DE, USA	[61]
*Brassaia actinophylla* (Umbrella Tree), *Hibiscus* *rosa-sinensis* (Tropical Hibiscus), *Pittosporum tobira* (Japanese Cheesewood)	100% kenaf supported normal growth but required more irrigation; blends with 70 to 80% kenaf plus peat and vermiculite produced shoot growth comparable to or better than two commercial mixes.	Weslaco, TX, USA	[4]
*Nephrolepis exaltata* (Boston fern), *Impatiens walleriana* (Impatiens), and *Viola* spp. (Pansies)	Pure kenaf led to slower growth than commercial mixes; 70% kenaf plus 30% peat produced plant growth comparable to commercial media.	Starkville, MS, USA	[23]
*Pinus halepensis* (Aleppo Pine)	Any mix containing kenaf core performed poorly relative to controls, indicating species-specific limitations for conifers under tested conditions.	Chalkidona, Greece	[62]
*Lactuca sativa* (Lettuce) and *Capsicum annuum* (Pepper)	Whole-stem kenaf with sand at sowing suppressed early growth even at 10 to 90 kenaf to sand; inhibition partly alleviated by ammonium nitrate soak; normal growth after transplant into kenaf-free media.	Athens, Greece	[64]
*Ilex crenata* (Japanese Holly) cv. Cherokee	Composted kenaf core compared favorably with pine bark, suggesting composting improves performance.	Poplarville, MS, USA	[65]
*Euphorbia pulcherrima* (Poinsettia)	In peat-based media, 50 to 70% coarse-ground kenaf by volume produced best growth and quality, comparable to a commercial control (Sunshine #1). Media with more than 70% fine-ground kenaf yielded smaller plants, leaf chlorosis, and container media shrinkage. Kenaf-amended media had lower water-holding capacity than the control and required more frequent irrigation.	College Station, TX, USA	[66]
17 vegetable and ornamental species	70% kenaf core plus 30% peat enhanced root mass; shoot dry weight higher in peat control for 7 species and comparable for 10; final height similar except tomato and *Celosia argentea* favored peat.	Starkville, MS, USA	[63]
Various ornamentals	Kenaf core substituted for pine bark and vermiculite; performance depended on grind fineness, kenaf proportion, and crop, indicating need to tailor particle size and blend ratio.	Lane, OK, USA	[22]

**Table 3:** Physical properties of ground kenaf core compared to peat and coco coir.

Growing Media	Bulk Density (g cm^−3^)	Total Porosity (%)	Available Water (%)
Ground Kenaf Core	0.06–0.15 [22,62]	90.7 [62]	41.9 [62]
Peat	0.04–0.2 [52]	83.8–96.4 [52]	6.1–47.8 [52]
Coco Coir	0.04–0.08 [59]	85.5–89.5 [59]	19.9–37.8 [52]

**Table 4:** pH and EC of ground kenaf core compared to peat and coco coir.

Growing Media	pH	EC (dS m^–1^)
Ideal	5.2–6.5 [25,30,31]	2.5–3.5 [25,34]
Ground Kenaf Core	6.03–7.17 [4,62]	3.18–4.7 [4]
Peat	3.9 [52]	0.2–0.49 [25,52]
Coco Coir	5.6–6.9 [52,59]	0.3–2.9 [59]

**Table 5:** Inorganic nutrient content of phosphorus (P), potassium (K), nitrogen (N), calcium (Ca), and magnesium (Mg) of ground kenaf core compared to peat and coco coir.

Nutrient	Ground Kenaf Core	Peat	Coco Coir
P (mg g^−1^)	0.14 [62]	0.25 [67]	0.27 [58]
K (mg g^−1^)	0.5 [62]	0.13 [67]	0.33 [58]
N (%)	1.44 [62]	0.0–5.0 [68]	0.44 [69]
Ca (mg g^−1^)	12.0 [62]	5.86 [67]	0.058 [70]
Mg (mg g^−1^)	2.34 [62]	0.97 [67]	0.055 [70]

**Table 6:** Cellulose, hemicellulose, and lignin content of ground kenaf core compared to peat and coco coir.

Growing Media	Cellulose (%)	Hemicellulose (%)	Lignin (%)
Ground Kenaf Core	32.1 [18]	41 [18]	25.2 [18]
Peat	5–20 [71]	10–30 [71]	5–40 [71]
Coco Coir	32–43 [18]	0.15–0.25 [18]	40–50 [18]

## Data Availability

The original contributions presented in the study are included in the article, further inquiries can be directed to the corresponding authors.

## Abbreviations

The following abbreviations are used in this manuscript:

CEC	Cation exchange capacity
EC	Electrical conductivity
